# Gut Microbiota and Alzheimer’s Disease: How to Study and Apply Their Relationship

**DOI:** 10.3390/ijms24044047

**Published:** 2023-02-17

**Authors:** Ngoc Minh Nguyen, Jungsook Cho, Choongho Lee

**Affiliations:** Integrated Research Institute for Drug Development, College of Pharmacy, Dongguk University-Seoul, Goyang 10326, Gyeonggi, Republic of Korea

**Keywords:** gut microbiota, Alzheimer’s disease, microbiota-gut-brain axis, experimental models, therapeutic strategy

## Abstract

Gut microbiota (GM), the microorganisms in the gastrointestinal tract, contribute to the regulation of brain homeostasis through bidirectional communication between the gut and the brain. GM disturbance has been discovered to be related to various neurological disorders, including Alzheimer’s disease (AD). Recently, the microbiota-gut-brain axis (MGBA) has emerged as an enticing subject not only to understand AD pathology but also to provide novel therapeutic strategies for AD. In this review, the general concept of the MGBA and its impacts on the development and progression of AD are described. Then, diverse experimental approaches for studying the roles of GM in AD pathogenesis are presented. Finally, the MGBA-based therapeutic strategies for AD are discussed. This review provides concise guidance for those who wish to obtain a conceptual and methodological understanding of the GM and AD relationship with an emphasis on its practical application.

## 1. Relationship between Gut Microbiota and Brain Function

Gut microbiota (GM) refers to the microorganisms living in the gastrointestinal (GI) tract [1]. Primarily, they come from all three taxa of microorganisms, including Bacteria, Archaea, and Eukarya, with Bacteria being the most predominant. Among them, the most popular gut bacterial species consist of *Proteobacteria*, *Firmicutes*, *Actinobacteria*, and *Bacteroidetes* [2]. From the evolutionary perspective, the GM has fostered a mutually beneficial relationship with the host in several ways [3]. For example, GM plays a critical role in metabolism by producing enzymes and metabolic intermediates not synthesizable by the host. In addition, the GM resident on the enteric mucosal epithelium serves as an immuno-neutral zone to defend against the entry of pathogenic microorganisms into the host. Beyond its direct effects on host metabolism and immunophysiology, it seems to indirectly impact the communication between the GI tract and central nervous system (CNS) in both health and disease states. In particular, GM can influence the neural network controlling gut function through the intrinsic and extrinsic nervous systems, such as the autonomic nervous system, enteric nervous system, and neuroendocrine and immune systems [4]. As a consequence, GM has been implicated in the regulation of neural development, neurotransmission, and the maintenance of brain homeostasis.

Considering the critical roles of GM in the normal neurophysiology of the host, some researchers suggested its direct involvement in the development of various brain diseases under pathogenic conditions. The current predominant theory postulates that the mechanism of GM-mediated neuropathogenesis involves GM-induced enhanced neuroinflammation and imbalanced inhibitory/excitatory neurotransmission. In this theory, GM dysbiosis, which is defined as a pathogenic imbalance in the gut microbial community, can upregulate local and systemic inflammations by secreting several bacterial endotoxins, typically lipopolysaccharides (LPSs). The translocation of these bacterially secreted LPSs from the gut to the bloodstream can lead to the so-called “leaky-gut” state, which is characterized by increased intestinal permeability, followed by infiltration of monocytes and secretion of neuroinflammatory cytokines and chemokines [5,6,7]. In addition, GM dysbiosis can also upregulate the microbial production of a wide range of neurotransmitters and neuromodulators, including dopamine, norepinephrine, serotonin, γ-aminobutyric acid (GABA), and short-chain fatty acids (SCFAs), as well as neurotoxic metabolites, such as D-lactic acid and ammonia [6,8,9]. Such GM-induced neuroinflammation and GM-derived neurochemicals can adversely affect host neural functions and inter-bacterial communication processes in a pathogenic manner, ultimately leading to various brain disorders [8].

To emphasize the critical roles of GM in the normal and pathological regulation of a variety of gut and brain functions, a new term, “microbiota-gut-brain axis” (MGBA), was defined, to describe the communication and influence between GM and brain. MGBA has been implicated in various CNS disorders such as depression, anxiety, autism, and Alzheimer’s disease (AD) [9,10,11,12,13]. Accumulating evidence suggests the direct involvement of GM in the regulation of social behaviors, stress resistance, and cognitive functions [5,14,15,16]. In particular, a number of studies suggested the pathogenic roles of GM in the development of Alzheimer’s disease, Parkinson’s disease (PD), and Huntington’s disease (HD) [17,18]. In regards to PD, alternation of GM was confirmed in PD patients by two separate studies [19,20]. Interestingly, the presence of α-synuclein-aggregates in the enteric nervous system before their appearance in the brain suggests their gut-to-to-brain spread theory [21]. In line with this, α-synuclein-mediated motor deficits were aggravated by GM in the mouse model [22]. GM dybiosis was also confirmed in the mouse models of HD [23,24,25]. Based on these observations, already-known pathogenic mechanisms for various brain diseases have been re-evaluated in the context of this newly-found relationship between GM and the brain.

## 2. Gut Microbiota and Alzheimer’s Disease

**Figure 1:**
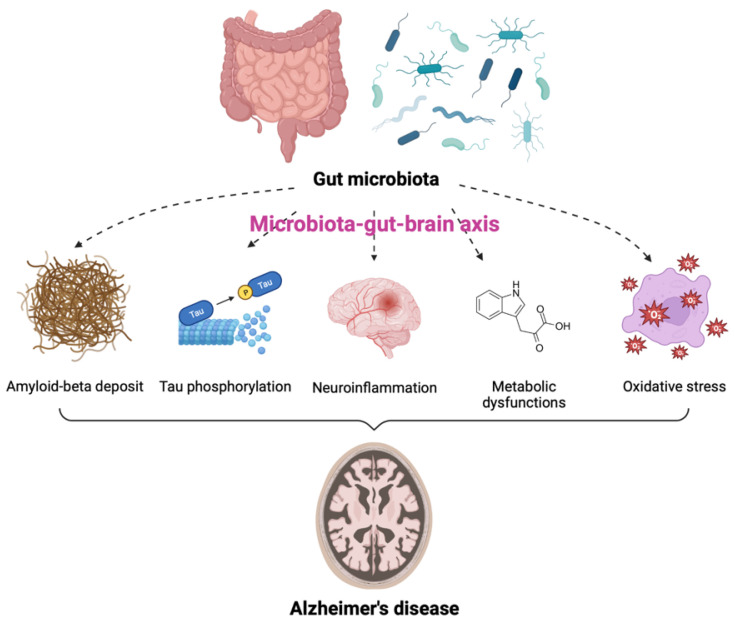
Contribution of gut microbiota (GM) to the pathogenesis of Alzheimer’s disease (AD). GM is involved in the onset and progression of AD through alterations of at least five pathogenic processes, including amyloid-beta deposit, increased tau phosphorylation, neuroinflammation, metabolic dysfunctions, and oxidative stress via the microbiota-gut-brain axis.

AD is one of the most common neurodegenerative diseases, characterized by a decline in cognitive function and neuronal loss. Neuritic plaques and neurofibrillary tangles (NFTs) are considered to be pathological hallmarks of AD [26]. Recently, numerous studies have described the notable impact of GM on the pathogenesis of AD [27,28,29,30,31,32]. In particular, GM dysbiosis was shown to have negative effects on brain function and host behavior through MGBA dysregulation, suggesting its potential role in the development of AD [33,34]. The GM is thought to be involved in AD through alterations of at least five different pathogenic processes. These include amyloid-beta (Aβ) deposit, increased tau (a microtubule-associated protein) phosphorylation, neuroinflammation, metabolic dysfunction, and oxidative stress (Figure 1). Individuals with GM dysbiosis due to intestinal diseases were also shown to be at high risk for developing AD [35,36]. Additionally, the degree of alteration in the fecal microbiome has been correlated with the severity of AD [37,38,39]. Approximately 85% of patients with dementia were reported to have alteration in GM compositions as compared to populations of healthy individuals [40] and decreases in GM richness and diversity were observed in patients with AD [30]. Moreover, the levels of the specific bacterial species were found to be correlated with the cerebrospinal fluid (CSF) biomarkers of AD pathology. These data indicate that the alterations in GM can be linked to neuropathological changes in AD [30].

Several GM species are associated with the production of amyloid fibers [41]. These amyloid fibers can cross the intestinal epithelium and blood-brain barrier (BBB) and induce several effects on the deposition of Aβ in the brain, resulting in AD pathogenesis [28,42]. Bacterial amyloids produced by GM promoted the misfolding of Aβ fibrils and oligomers [43]. In addition, due to the similarity in structure and immunogenicity, amyloids secreted by GM can bind to Toll-like receptor 2 (TLR2) on microglia and activate inflammatory responses [27]. These factors may enter the brain and directly affect AD pathology. Moreover, IL-17-expressing T helper cells migrate to the CNS via the gut-associated lymphoid tissue and interact with microglia, contributing to neurodegeneration in AD [27]. Furthermore, GM dysbiosis reduces the clearance of Aβ by affecting the gut mucosal barrier and energy homeostasis [44]. Collectively, GM increases the production of Aβ fibers, accelerates their aggregation and misfolding, and reduces Aβ clearance, all of which may contribute to the development and progression of AD.

Hyperphosphorylated tau and disintegrated microtubules are characteristics of NFTs, another pathological hallmark of AD [45]. A recent study found elevated levels of a GM-derived metabolite, trimethylamine *N*-oxide (TMAO), in the CSF of patients with AD dementia [46]. TMAO was involved in tau pathology, suggesting the influence of GM in AD pathogenesis through tau dysregulation [46]. Other researchers revealed GM-induced hyperphosphorylation of tau through the activation of the glycogen synthase kinase 3 beta (GSK-3β) pathway, resulting in cognitive impairment [47]. Moreover, another study found alleviation of Aβ deposition, tau pathology, and memory impairment following fecal microbiota transplantation (FMT) from healthy wild-type mice into transgenic (Tg) mice with AD-like pathology, including amyloid and NFTs [48]. Although the comprehensive tau-mediated AD pathogenesis by GM remains elusive, these studies indicate that the modulation of GM may be a potential strategy to alleviate tauopathy for AD treatment.

Neuroinflammation is one of the plausible mechanisms to explain AD pathogenesis. This physiological response to stimuli maintains homeostasis, but excessive inflammatory responses cause harmful effects on the CNS. Changes in GM composition can trigger peripheral immune responses by activating immune components and regulating the levels of pro-inflammatory cytokines in the brain [49,50,51]. Recent evidence suggests that GM dysbiosis is associated with the development of AD through neuroinflammation [52,53,54]. The increase in pro-inflammatory GM is accompanied by enhanced systemic inflammation and neuroinflammatory processes. Since GM dysbiosis can lead to defects in the activities of microglia, its activation can contribute to the progression of AD. Several gut microorganisms were reported to produce nitric oxide (NO) and activate microglia, thereby exacerbating the AD condition [55].

Metabolic dysfunction is also another hallmark of AD. As mentioned above, GM can produce bioactive metabolites, such as SCFAs and neurotransmitters, which can modulate the immune system and influence brain activities [56,57]. These metabolites can cross the BBB to affect cognition directly or indirectly through immune, neuroendocrine, or vagal mechanisms [58,59]. A meta-analysis of observational studies reported that increased levels of GABA were associated with a lower risk of AD [59]. There is a significant difference in the level of GM metabolites between AD patients and the control group [60]. Several metabolites upregulated by GM in patients with AD, including indole-3-pyruvic acid, an endogenous metabolite of tryptophan, and SCFAs, were correlated with cognitive impairment [60]. Interestingly, PD patients showed the opposite phenomenon, which is the reduction of SCFAs [61]. In addition, serotonin concentrations in the serum and urine of AD patients were significantly lower than in controls [62]. Most GM-associated metabolic pathways have been predicted based on sequencing analysis of the microbiome [63]. Therefore, validation is essential to prove the relationship between certain GM and AD pathogenesis.

Oxidative stress is another factor responsible for AD pathology. GM dysbiosis can involve in AD development by influencing the levels of oxidative stress in the CNS. For example, NO conversion from nitrate and nitrite by *Lactobacillus*, *E. coli*, and *Bifidobacterium* increases the permeability of the BBB and contributes to neurotoxicity in AD [64,65]. Increased intestinal permeability and following GM dysbiosis were also confirmed in the mouse model of HD [66]. Pathogenic enteric bacteria, such as *Salmonella* and *E. coli*, can induce hydrogen sulfide production in the gut, resulting in decreased mitochondrial oxygen consumption and overexpression of pro-inflammatory cytokines [67]. Hydrogen is a highly diffusible bioactive gas produced mainly by anaerobic cocci, which belong to the *Enterobacteriaceae* family [68]. GM dysbiosis may lead to reduced hydrogen production and limit gas supply to neurons in the CNS. Taken together, alterations in the GM favor oxidative stress, resulting in the pathogenesis of AD.

## 3. How to Study the Relationship between GM and AD

**Figure 2:**
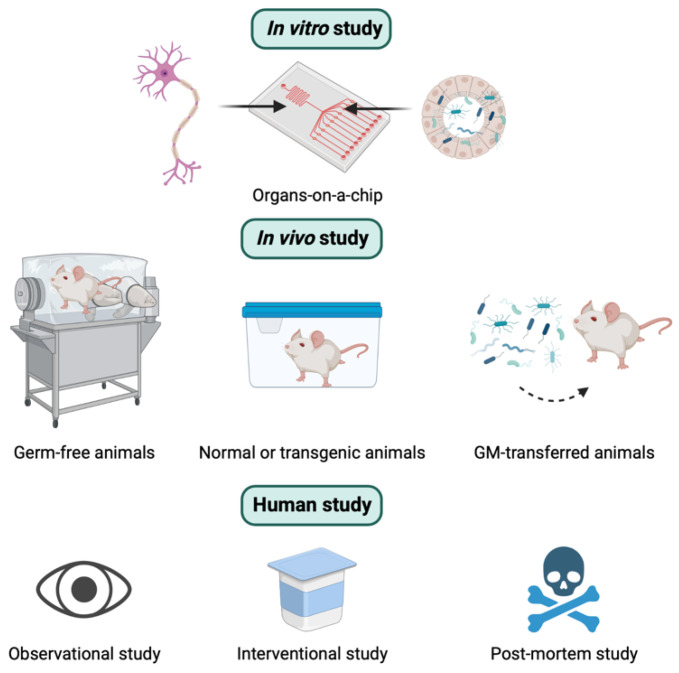
Three different types of experimental approaches to studying the role of the microbiota-gut-brain axis in the pathogenesis of Alzheimer’s disease. See the text below for the details.

Overall, similar to other research, there are three different types of experimental approaches used to explore the relationship between GM and AD. These include in vitro, in vivo, and human studies (Figure 2). In this section, the concepts, strengths, and weaknesses of these three methods to study the role of the MGBA in the pathogenesis of AD are presented.

### 3.1. In Vitro Study

Although conceptually possible, cell-based studies have several limitations in studying the interconnection between GM and AD. The study of the MGBA has almost exclusively relied on the genomic or metagenomics analysis of samples collected from animal or human models. Up to now, there is a lack in in vitro models to simulate the communication between GM complex and intestinal epithelium or host-microbiome interactions [69,70]. To discover the disease status or intervention related to GM thoroughly, there is a need for longitudinal studies in animals and humans, which are difficult, expensive, and time-consuming. Therefore, this review will focus on in vitro gut fermentation models and their application for longitudinal studies to supplement in vivo microbiome studies.

To study the role of GM in the pathogenesis of AD, technology has proved a powerful tool to support in combination with cellular biology. Organ-on-a-chip (OOC) technology is a typical example of this combination [71,72,73]. This design successfully integrates the physiological features [70] in a model with bacterial molecular flux, microbial co-cultivation at the anoxic–oxic interface, a mucus bilayer with physiological thickness, or physical deformations for peristalsis-like motion [74,75]. However, most of today’s in vitro gut models are designed without the presence of tissue-resident immune cells [76]. The inclusion of the typical GM and human immune cells offers a promising gap to develop a reliable and predictive in vitro model to elucidate the relationship between GM and AD.

In the in vitro three-dimensional (3D) models used in AD studies by Sommer, brain cells were loaded in a suitable hydrogel matrix and cultured in a novel microfluidic device under perfusion with flow rates similar to the interstitial flow in brain tissue [70]. An innovative microfluidic device constitutes the building block of a multi-organ platform, and a hydrogel-based 3D model of brain cells can be housed in the microfluidic device to facilitate the expression of APP and accumulate amyloid, which is related to AD pathology. Based on this feature, the device can host suspended 2D and 3D cell-based models cultured alone or interconnected with other OOC units, to represent biological barriers, such as the BBB. Human neuroglioma H4-SW cells were chosen to test this model for their simplicity and capability to produce high levels of toxic Aβ. In particular, neural progenitor cells were cultured in a microfluidic chip under continuous flow conditions and a gradient of oligomeric assemblies of Aβ [77]. The main drawback of this study is the static conditions, although it provided evidence about the amyloid hypothesis in AD. Therefore, it is important to improve the dynamic conditions in in vitro studies for the simulation of AD pathogenesis.

Microfluidic OOCs in vitro modeling is a successful production of combining cellular biology and technology. This model provides an extraordinary opportunity to study the MGBA mechanisms in the development of neurodegenerative disorders, including AD. However, there is room left to improve more reliable and physiologically relevant OOCs. Currently, the most challenging aspects are building a dynamic environment and co-culture of multiple cell populations in multi-stage OOCs [78].

### 3.2. In Vivo Study

In vivo studies play a major role and provide a powerful tool for studying the underlying pathogenic mechanisms of GM-mediated AD. Various animal models, such as mice, rats, pigs, and zebrafish, have been used for GM research [79,80,81,82]. Furthermore, inbred and knockout techniques are regularly used for some animals like mice or rats [83,84]. There are different types of animal models depending on the origin of GM. They can be germ-free (GF) models (animals without microorganisms living in them), conventionally raised animals (animals colonized with their native GM), gnotobiotic animals (animals colonized with specific microbe(s), or human GM-transferred animals (animals colonized with human GM) [85]. These models are illustrated in Figure 2. The major advantage of animal models is the availability of pharmacological interventions that are not approved for humans and the access to organs and tissues after scarification [84]. The advantages and disadvantages of in vivo animal studies are summarized in Table 1.

Among different types of animal models, the GF model is the most popular one to study GM-host interactions. Almost human microbiota can be colonized in GF mice, while it is difficult to transfer predominant taxa in the gut of the human to ordinary mouse gut. In several cases, after colonization, the growth of microbiota may be modified and loss the characteristics of human donors [87,88]. Similarly, GM composition is likely to be altered by the disease states of GM dysbiosis after the colonization of GF mice, which may no longer reflect the composition in the disease state [89]. Several factors including diet, housing conditions, and sample collection procedures are also known or suspected to alter GM in animals [90]. Furthermore, origins or commercial suppliers of animal models are other factors to affect the variety of GM composition [83,91,92,93]. Despite the huge advantages and wide application of in vivo research, there are still other disadvantages of animal studies. For example, the reduction of animal experiments is necessary for ethical reasons. In addition, animal models are economically more burdensome than most in vitro models. On top of this, they need appropriate and special animal housing facilities, including GF facilities [84]. Moreover, longitudinal studies with animals are expensive and challenging since numerous animals need to be sacrificed at each time point [94,95]. Ultimately, there is a significant gap in the GM and immune systems between humans and animals, making it difficult to translate the results of in vivo research to human clinical trials [96].

The use of GF animals has been instrumental in understanding microbe-host relationships [97]. The first GF model in rodents was successfully generated in the 20th century [98]. Similar methods are now typically used to produce many generations of GF animals. Cesarean section is preferred to avoid inoculation of pups by microbiota [99,100]. Regular examinations of cages and feces are carried out to confirm the absence of bacteria [101]. Subsequent GF animals can be bred in an isolator, and GF pups can be born virginally. Alternatively, an embryonic transfer can be performed in GF animals into a GF host mother at the two-cell stage [101]. GF animals have shown completely different developmental and physiological processes when compared with animals hosting commensal bacteria. GF animals are lighter and live longer. They have reduced levels of most GI luminal amino acids when compared with specific pathogen-free mice [100,102,103]. The lack of commensal microbes renders GF animals have an abnormal impact on immune systems, hormone signaling, metabolism, and neurotransmission [104,105,106]. Interestingly, phenotypes of GF animals are different across species, sex, research group, and even strain. This indicates the importance of microbiota and host genetics in determining several animal phenotypes [107,108].

Despite its many strengths, GF mice have several limitations. They include physiology, neurodevelopment, and immunity that are aberrant from human counterparts. All these factors limit translatability to clinical applications [86]. Nonetheless, GF mice have been used as the first of choice to investigate the involvement of microbiota in a given process [109,110]. Moreover, the results of GF studies begin to be applied to other non-rodent species such as pigs to maximize their translational value [109]. Alternatively, the transfer of mice with specific, known strains of bacteria (gnotobiotic animals) has also been utilized to investigate the specific microbiota-host relationship [111]. Among such methods, the altered Schaedler flora (ASF) mouse line has been the most widely used [112,113]. Eight bacterial colonization used in ASF mice simplified the study of microbiota involvement in brain diseases. A study with ASF mice was also able to produce more clinically relevant data than GF studies. When performing the experiments using GF mice, several host developmental defects including an underdeveloped immune system, slower intestinal epithelial turnover, differing nutritional requirements, and less body fat were frequently found. ASF mice turned out to be able to reduce these limitations [114]. Therefore, the ASF model is regarded as an attractive alternative to studying the effects of GM on stress-related brain disorders [112].

In the below paragraphs, illustrations and descriptions are mentioned about the utilization of animal models. To elucidate the impact of GM manipulation on AD pathology, a study used the 5xFAD model mice treated with antibiotics or probiotics [115]. While antibiotics treatment significantly reduced viable commensals, probiotics treatment transiently increased *Lactobacillaceae*. An analysis of Aβ deposition in the hippocampus confirmed the finding of ameliorated pathology. This study provides evidence that antibiotics might elicit a beneficial effect on AD pathology by the subsequent decrease in the Aβ influx. Another study also used the 5xFAD Tg mice model to understand the role of commensal gut bacteria on the progression of cognitive decline in AD [116]. The oral administration of *Bifidobacterium* brought alterations in the GI tract related to AD pathogenesis. These alterations included changes in GM composition, reduced fecal and blood LPS levels in feces and blood, suppressed nuclear factor kappa B (NF-κB) activation, and tumor necrosis factor-alpha (TNF-α) expression in 5xFAD Tg and aged mice. These results suggest that gut dysbiosis and excessive endotoxin production can lead to endotoxemia and systemic inflammation, and CNS disorders. Moreover, the administration of *Bifidobacterium* was able to suppress the GI inflammation, resulting in the attenuation of cognitive decline in AD and aged mice through the regulation of neuroinflammation by the MGBA.

Various AD mouse models have been used to investigate the correlation between MGBA and AD. The D-galactose (D-Gal)/AlCl_3_-induced AD mouse model was used to examine the effect of the water extract of *Gastrodia elata* rhizoma (WERG) on MGBA in AD treatment. WERG treatment enriched the gut probiotics and decreased the levels of phosphorylated tau, therefore, the cognitive impairment of D-Gal/AlCl_3_-induced mice was improved in the WERG-treated group [117]. The regulation of GM by gastrodin (Gas) from *G. elata* for neuroprotection in AD was also determined using a D-Gal–induced AD model [118]. In that study, Gas was found to mitigate the memory dysfunction of AD mice. Interestingly, the antibiotic cocktail partially reversed the neuroprotective effect of Gas by changing the GM composition. In conclusion, Gas could improve the memory function of AD mice by partly targeting the MGBA and mitigating neuronal inflammation [118].

In addition to AD mice models, ordinary mice models are also used in the study of GM and AD. In research assessing the effect of GM depletion on anxiety- and depression-related behaviors, C57BL/6 mice were treated with an antibiotic cocktail for a long period of time from weaning to adulthood [119]. Results showed that there is a significant decrease in anxiety-like behaviors in the healthy antibiotic-treated group. Antibiotic treatment from early adolescence prevented the development of anxiety- and depression-related behaviors in AD-induced mice. In another study investigating the effect of *Lactobacillus* dominance by Korean red ginseng on the improvement of AD, Tg mice (Tg2576) were used for the experimental model of AD [120]. It was found that Korean red ginseng improved the cognitive behavior of mice and decreased the Aβ42/Aβ40, indicating reduced Aβ accumulation. In particular, the diversity of GM was altered, showing the increased population of *Lactobacillus* species.

Interventions, such as FMT, were introduced to in vivo studies to prove the effect of GM on the pathogenesis and treatment of AD. Research with an APPswe/PS1dE9 Tg mouse model was used to evaluate the efficacy of FMT for AD treatment [121]. FMT treatment improved cognitive deficits and reduced the brain deposition of Aβ in APPswe/PS1dE9 Tg mice. FMT treatment reversed the changes in the GM and SCFAs. In another study, FMT was proved sufficient to induce behavioral phenotypes in GF mice [122]. Kundu et al. used C57BL/6 mice to study FMT for AD treatment [122]. In their study, FMT transferred from 5xFAD mice to normal C57BL/6 mice decreased hippocampal neurogenesis and brain-derived neurotrophic factor expression, resulting in memory decline. This result can clarify the role of 5xFAD mouse-derived microbiota in AD development.

### 3.3. Human Study

GM can be studied in humans with a wide variety of individuals, including healthy volunteers, patients with a disease, patients with ileostomies, and individuals with sudden death [84]. The major advantage of human studies of GM is biological significance. Several antibiotics, including cefepime [123], amoxicillin [124], rifampicin [125], D-cycloserine [126], and doxycycline [127], reduced Aβ pathology and improved cognition in clinical studies. Furthermore, live and post-mortem studies using plasma [128] and brain samples [129,130,131] further indicated the greater LPS abundance in patients with AD compared with control individuals. However, it seems to be very hard to draw solid mechanistic conclusions based on these results because we cannot distinguish the direct or indirect effects of pharmacological interventions on the structure or functionality of the GM [95]. On top of this, additional challenges need to be overcome in human microbiome studies. For example, stringent ethical requirements for human study need to be satisfied beforehand. This may include modification of the research protocols and the prohibition of the usage of uncharacterized compounds [84]. In general, the time-series measurement methods used in human studies are costly and time-consuming. Extensive clinical data need to be provided to distinguish whether GM variability comes from pharmacological intervention or not. Moreover, due to higher percentages of withdrawal in long-term studies, researchers must consider patient numbers, which should be great enough to draw statistically meaningful conclusions [84]. Cohort variability due to host heterogeneity is another challenging factor to control in human studies [132,133]. Interindividual baseline variation of the GM with different responses to the same treatment is an additional confounding aspect to correlate the changes in the GM to the experimental intervention [134]. For example, the fecal microbiota transfer to different individuals exhibited different responses to the ciprofloxacin treatment [134,135]. Even the repeated administration of ciprofloxacin in the same patient also demonstrated different responses due to the composition change of the GM before and after drug treatment [134]. Several improvements to current research methodologies have been proposed to address these challenges associated with GM human studies. Stratification of study participants based on baseline GM may be helpful for better identification of GM alteration after drug administration. Diet has emerged as one of the most important factors responsible for normal variation in GM, as the relative composition of GM is strongly influenced by nutrients [136]. However, the data from properly controlled dietary intervention studies are very hard to interpret. Walker et al. studied the fecal microbiota of 14 overweight men who consumed a precisely controlled diet for 10 weeks [137]. While there is a dramatic increase in certain bacteria after starting the diet, they found that fecal microbiota clustered more closely by individuals than by diet. Patient compliance and the accurate follow-up of food consumption by study participants are other important factors to determine the quality of these types of studies. The source of the samples needs to be diversified in data analysis since microbial communities found in feces are not representative of the whole GI tract [94,138,139]. If researchers are interested in the interaction between bacteria and the gut mucosa, the collection of intestinal biopsies may be more desirable.

Several studies have reported that GM composition is related to AD pathology, but the observed differences are inconsistent across studies. An observational study in the Netherlands investigated the associations between GM composition and AD biomarkers using machine learning models [140]. Results showed that GM composition was associated with amyloid and p-tau status, which were the two characteristics of AD. Another study, which was conducted in China, examined the structural and functional dysbiosis of GM in AD patients [38]. The data demonstrated a remarkable reduction in bacterial diversity and alterations in the taxonomic composition of the fecal microbiota of patients with AD. The study established the structural and functional dysbiosis of fecal microbiota in AD patients. The results further suggest the potential for the use of gut bacteria for early diagnosis of AD and personalized treatment for patients with AD.

To thoroughly understand the relationship between MGBA and AD, a study of the microbial-derived metabolite is another approach. TMAO, which is generated by choline metabolism, is a known risk factor for AD [46]. TMAO was found to be higher in the CSF of individuals with AD dementia than in the control group. In addition, elevated TMAO in the CSF was associated with p-tau and p-tau/Aβ42, as well as neuronal degeneration. These findings provide additional insight into the involvement of GM in AD [46].

Several interventional human studies were conducted regarding the role of GM in AD pathogenesis and treatment, including case reports and clinical trials. A case report about cognitive function improvement after FMT in patients with AD dementia in 2021 provided further knowledge about this subject [141]. In that study, the cognitive function showed an improvement after FMT intervention based on the test score. The intervention also changed the GM composition in the recipient’s feces. This finding suggests a relationship between GM and cognitive function in AD. Furthermore, it also suggests a novel therapeutic option, FMT, for patients with dementia [141]. Another case showed a rapid improvement in AD symptoms following FMT [142]. The patient reported improvements in mental acuity. These findings supported the central role of GM in neurological dysfunctions, such as AD [142].

A randomized multicenter trial was conducted to examine the effects of probiotics on cognition and mood in the elderly [143]. The results showed that the probiotics group had greater improvements in the mental flexibility test and stress score than the placebo group. Probiotics change the composition of GM, promote mental flexibility, and reduce stress in healthy older adults. These results support the hypothesis that probiotics provide health-promoting properties as a part of a healthy diet in older adults [143]. Another explorative intervention study aimed to examine the effect of probiotic supplementation in patients with AD dementia [144]. The results showed that supplementing patients with a wide variety of probiotics affected not only tryptophan metabolism in serum, but also GM composition [144].

Although there is an increase in advanced human studies of MGBA and AD, there is still a gap in the application of knowledge about MGBA in AD therapies. Further human research, especially interventional studies, should be conducted in the future to build a concrete conclusion about the role of GM in AD pathogenesis and provide helpful evidence for GM-related treatments in AD.

### 3.4. Relevant Techniques

Elucidation of GM composition is necessary to study the relationship between GM and AD [145]. The 16S rRNA gene sequencing has been the main technique in GM analysis for decades. This technique is applied in both in vivo studies and human studies [119,141,146,147]. Fecal samples are usually collected to investigate the GM profile, total DNA is then isolated, measured, and 16S rRNA gene sequencing is performed [148].

To determine the diversity and richness of GM, alpha diversity is usually represented by indexes such as Shannon, Chao1, and Simpson. Meanwhile, beta diversity is usually represented by Principal Coordinate Analysis (PCoA) for the differences in composition between groups [118]. While alpha diversity is a measure of microbiome diversity applicable to a single sample, beta diversity is a measure of the similarity or dissimilarity of two communities. Furthermore, LEfSe analysis is used to identify the specific individual bacterial taxa which cause the differences between groups. LEfSe analysis refers to the LDA (Linear discriminant analysis) Effect Size analysis. LEfSe is used to discover high-dimensional biomarkers and reveal genomic characteristics, including genes, metabolism, and classification [118,146].

Recently, high-throughput sequencing of the full 16S gene has been applied widely to investigate the relationship between GM and AD [149,150,151]. Low-throughput sequencing methods used the polymorphisms within the gene to distinguish strains or subspecies. Meanwhile, the complete full-length 16S gene is sequenced in a high-throughput method. The full 16S gene sequencing provides better taxonomic resolution, and real and significant advantages over sequencing commonly targeted variable regions [152].

## 4. GM-Directed Therapeutic Options to Ameliorate the Progression of AD

**Figure 3:**
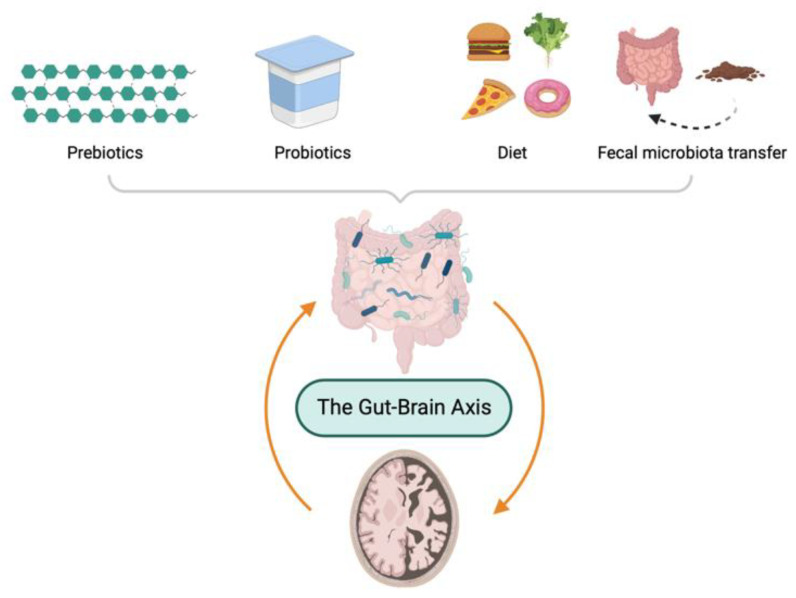
Gut microbiota-directed therapeutic options to ameliorate pathology of Alzheimer’s disease.

Due to the importance of maintenance of a healthy microbiota, several modulators of GM have been proposed such as microecological regulators, including prebiotics and probiotics, dietary intervention, and FMT. In this section, as depicted in Figure 3, the therapeutic options based on GM for the treatment of AD are discussed.

### 4.1. Prebiotics

Prebiotics are defined as non-digestible short-chain carbohydrates that possess the ability to change the composition and metabolism of GM beneficially [153]. They can act as specific fermentation substrates for SCFA-producing probiotic genera, thus affecting both GI and extra-intestinal functions [154]. Recent studies in animal models and humans have shown possible effects on psychiatric symptoms [145]. Some promising results have been reported regarding the use of prebiotics for the prevention or treatment of AD [145,153]. The administration of yeast beta-glucan to mouse models of AD is effective in restoring the balance between pro- and anti-inflammatory GM species [146]. Lactulose, which was the first commercially available prebiotic, was found to improve short-term memory and learning retrieval in AD mice [147]. In addition, mannan oligosaccharide treatment for 8 weeks induced the growth of *Lactobacillus* species and decreased *Helicobacter* abundance, resulting in reduced LPS leakage and BBB dysfunctions in 5xFAD Tg mice [63]. Interestingly, GM reconstitution by this prebiotic was also accompanied by decreased Aβ accumulation, restoration of redox homeostasis, and increased butyrate levels [63]. Similar results were seen in rodent models of AD treated with *Marinda officinalis*-derived oligosaccharides, with the effect of improved memory and learning ability [36,148]. Although the mechanism of action remains to be elucidated, prebiotic administration may be beneficial in the treatment of AD [148,149]. Moreover, a combination of probiotics and prebiotics (or synbiotics) seems to be more effective in increasing neurogenesis and reducing neuroinflammation as compared to prebiotics alone [149].

Daily administration of fructan, a well-known prebiotic, reduced the risk of AD development, as data from a large longitudinal study in older adults [150]. However, other authors suggest that more evidence for the use of prebiotics in clinical practice is still needed for concluding the normalization of several factors such as age, gender, ethnicity, and diet [151]. In conclusion, prebiotics may be helpful as a preventive or therapeutic therapy for AD, and there is a need for more human trials to concrete the importance of prebiotic treatment.

### 4.2. Probiotics

Probiotics are living microorganisms that are beneficial to the host with an adequate amount [152]. Probiotics have recently gained attention in brain function because they improve GM by positively influencing the MGBA. They are also known as psychobiotics because they attempt to restore the imbalances in the MGBA. They can release neuroactive substances and directly affect the human brain [155,156,157]. Many of these responses arise from the regulation of intracellular signaling pathways, such as mitogen-activated protein kinases (MAPKs) and NF-κB [158]. Several studies have shown that probiotic supplementation can restore the GM, improve the integrity of the gut barrier and BBB, and reduce neuroinflammation, as well as cognitive decline [159]. Administration of probiotics increased *Actinobacteria* and *Bacteroides* species in the GM composition of AD animal model, significantly affecting long-term memory, inflammation, and neural plasticity [160]. Recently, a study on Tg AD mice demonstrated that the administration of a probiotic formulation significantly reduced oxidative stress by inducing sirtuin-1-dependent mechanisms [161]. In addition, the probiotics from a mixture of *Lactobacillus* and *Bifidobacterium* modified specific neurotransmitters, such as GABA and glutamate [162]. In another work, short-term administration of *Bifidobacterium breve* strain A1 suppressed immune response and neural inflammation in Aβ-injected mice [163]. Furthermore, a mixture of *Lactobacillus acidophilus*, *Lactobacillus fermentum*, *Bifidobacterium lactis*, and *Bifidobacterium longum* improved learning disability and oxidative stress of rats intra-hippocampally injected with Aβ1–42 [164]. By using in vitro Caco-2 cell monolayer, di Vito et al. confirmed the modulation of tight and adherent junction and prevention of LPS-induced inflammatory damage by administration of commercially available probiotic formulation [165].

In a recent randomized trial, 60 patients with AD were divided into two groups and administered milk (control group) or probiotics (probiotic group). After 12 weeks of daily administration of probiotics, a significant improvement in the mini-mental state exam score was reported in the treated group, as compared to the control group [166]. Similarly, data from another meta-analysis reported a significant amelioration in cognition and a consistent reduction in post-intervention levels of malondialdehyde and high-sensitivity C-reactive protein in subjects receiving probiotics. These results indicate that probiotics, even when taken alone or in a combination supplement, have shown great potential in the reduction of AD progression. However, the appropriate strains, doses, time of treatment, routes of administration, and safe use of probiotics for AD need to be studied in the future.

### 4.3. Diet

Interactions between diet, GM, and host are important factors influencing health. Diet is one of the major factors involved in shaping GM composition [167,168]. Based on recent evidence, the Mediterranean diet (MD) and the ketogenic diet (KD) are likely to be the most promising dietary therapies for AD.

MD is a way of eating based on the traditional cuisine of countries bordering the Mediterranean Sea. This diet is characterized by a high intake of fruits, vegetables, cereals, and legumes; and a low intake of meat, high-fat dairy, and sweets. It is considered to be an anti-inflammatory diet and prevents the occurrence of several chronic diseases [169,170]. MD is also associated with a lower risk of AD [171]. Two large randomized controlled trials have demonstrated a positive correlation between “MD plus olive oil” or “MD plus nuts” with cognitive performance [172,173]. Recently, another clinical study linked MD with improved cognition [174]. Furthermore, a narrative systematic review and meta-analysis demonstrated a protective and likely therapeutic role of MD in AD and confirmed its ability to prevent cognitive impairment [175]. In general, a dietary pattern rich in fruits, vegetables, and legumes and low in saturated fats and sweets seems to provide protective effects [176]. Similarly, results highlighted the benefits of MD as a protective factor against AD [177]. Possible neuroprotective mechanisms common to these diets include the presence of antioxidants and anti-inflammatory compounds that help reduce inflammation and oxidative stress in the brain, and high levels of fiber, vitamin C, β-carotene, and folate. As a result, it improves brain integrity and increases the amount of brain tissue [178]. It has also been reported that saturated and trans fatty acid deficiency may reduce BBB dysfunction and amyloid aggregation [179,180].

KD is a term for a low-carbohydrate and adequate protein diet [181]. Recent studies have demonstrated a role for KD in the compositional remodeling of GM, thereby promoting its protective effects in various CNS disorders, including AD [182,183]. When sugar is in short supply, ketone bodies, which are used as alternative energy substrates for glucose in many organs, including the brain, are produced to break down and oxidize fat [184]. In mice models, ketone bodies have been demonstrated to influence neurotransmission, reduce neuroinflammation and oxidative stress, as well as reduce amyloid accumulation, and improve learning and memory abilities [185,186]. In humans, KD may benefit people with mild cognitive impairment or AD [183,187]. Similar to the mechanism (ketone body production), the medium-chain triglyceride diet/supplementation and modified Atkins diet are effective not only for symptoms such as fatigue and daytime sleepiness in Parkinson’s disease but also for cognitive decline in AD, epileptic seizures, and mood swings in depression [187,188]. Additionally, the modified Atkins diet, which does not restrict protein intake as the KD diet, allows much more flexibility than the classic KD. Overall, dietary patterns that lead to ketone production appear to represent a promising treatment for AD, although they reveal human protective mechanisms and adverse effects such as inflexibility and variability in dietary plans. To do so, more research is needed. It can easily lead to school dropouts and a lack of plant foods rich in vitamins and other antioxidant compounds [182].

In summary, dietary interventions are generally safer and more beneficial than drug therapy because they are inexpensive, easy to administer, and reduce the burden on caregivers of AD patients.

### 4.4. Fecal Microbiota Transplantation

FMT is a procedure that transfers the healthy donor’s GM to the recipient for therapeutic purposes. It is considered a safe procedure with minor and transient side effects [189]. It has been shown to be effective in the treatment of recurrent *Clostridium difficile* infections [190]. To date, most of the studies have been conducted in animal models, with promising results but a concrete conclusion has not been drawn yet.

Transplantation of feces from AD model donor mice into healthy mice resulted in impaired neurogenesis, increased memory impairment, increased circulating inflammatory cytokines, and Aβ plaque deposition [191,192]. Similarly, GF mice that received feces from APP/PS1-Tg mice that developed brain Aβ deposition showed increased plaque formation [49,193]. Furthermore, FMT from AD patients to GF mice accelerated cognitive decline and the reduction of microbiota-derived metabolites important for nervous system function [193]. Another study confirmed improved cognition, decreased amyloid accumulation and tau expression, improved synaptic plasticity, and increased SCFA-producing gut bacteria [194]. FMT effectively restored the microbiota composition in the APP/PS1 Tg mouse model of AD, improving the conditions of microglia and Aβ deposition [194].

In terms of human studies, two case studies are showing promising results [141,142]. Hazan demonstrated an improvement in AD symptoms (including cognitive function, memory, and mood) in a man aged 82 after FMT from the recipient’s wife [142]. In a second case study, a woman aged 90 with AD and severe *C. difficile* infection who underwent FMT from a healthy young donor showed improvements in cognitive function, GM composition, and SCFA production [141]. Interestingly, FMT also improved GM dysbiosis and cognitive deficits in the mouse model of HD [195].

These studies demonstrate that FMT can rapidly and effectively restore GM dysbiosis and brain dysfunction in patients, suggesting that restoration of GM homeostasis by FMT may have beneficial effects on AD treatment. However, several limitations remain for its wide application, such as standardization of the procedures, timepoint, and treatment period, as well as inclusion criteria of donor and recipient [196,197,198]. Therefore, more human trials will be conducted in the future to provide evidence for the efficacy of FMT and optimize the intervention.

## 5. Conclusions

The MGBA is an enticing target to understand the pathogenesis of AD, as well as to develop new therapeutic options to prevent and treat this disease. The MGBA can influence the development and progression of AD through various pathways, from Aβ deposition and tau phosphorylation to neuroinflammation, metabolic dysfunction, and oxidative stress. Numerous methodologies using in vitro cell models, animal models, and humans have been developed to gain insight into the relationship between GM and AD. Understanding of normal and pathogenic roles of the MGBA in host neurophysiology is critical for the development of mechanism-based prophylactic and/or therapeutic strategies for AD.

## Figures and Tables

**Table 1:** The advantages and disadvantages of animal models in studying the effects of gut microbiota on Alzheimer’s disease [86].

Advantages	Disadvantages
Allowance of experiments that are not permissible in humans due to ethical reasons Use of numerous genetically modified and knockout mouse models Less burden on maintenance cost, high reproduction, and short life span Homogenous genetic background and enhanced reproducibility of experiments Control of sources of variations, minimizing unwanted environmental noises to GM	Fundamentally different physiology from humans in animal models Alteration of GM composition in different animal models No human-specific crosstalk between the GM and the host in animal models No inherent genetic variations in animal models Inability to recapitulate “real” human-GM relationship in animal models

## Data Availability

Not applicable.
